# Two-Step Converging Spherical Wave Diffracted at a Circular Aperture of Digital In-Line Holography

**DOI:** 10.3390/mi13081284

**Published:** 2022-08-09

**Authors:** Peng Tian, Liang He, Xiaoyi Guo, Zeyu Ma, Ruiqi Song, Xiaoqiao Liao, Fangji Gan

**Affiliations:** School of Mechanical Engineering, Sichuan University, Chengdu 610065, China

**Keywords:** converging spherical wave, digital in-line hologram, single exposure

## Abstract

The aspheric light emitted from a pinhole restrains the reconstruction quality of a digital in-line hologram. Herein, the Fresnel-diffracted spot from the first step converging spherical wave diffracted at a rough circular aperture is collimated and expanded to generate an even plane wave, which is converged again by an objective lens and matching a minimum aperture while the central spot is varying from light to dark. We observed that the collected background hologram is filled with a round spot with high contrast as an ideal spherical wave. The resolution board and biology experimental results demonstrated a distinctively reconstructed image without any image processing in a single exposure. The adjustable field of view and magnification, single exposure, and noncontact make it suitable for an online microscope.

## 1. Introduction

Digital in-line holography (DIH) using a spherical wave as the illuminating source, diffracted via a pinhole, is a facile realization strategy of holographic microscopy [1,2]. The object is placed behind the pinhole with a short distance, and the scattered beam of the object forms the interference fringes with the un-scattered reference beam, considered as an ideal spherical wave. The tested object’s image is reconstructed by numerical calculation of wave propagation. At the range of visible spectrum, DIH yielded three-dimensional (3D) images of objects with micrometer resolution, applied widely in biology and medical microscopy [3,4,5], lab-on-a-chip analysis [6], and particle tracking [7,8,9], etc.

In pinhole holographic reconstruction, the reference light is usually regarded as an ideal spherical wave, which affects directly the quality of the reconstructed image, such as blur and shadow. Although the size of pinhole and wavelength are alternative, machining errors of pinhole and uncertain roundness could not be avoided. They will lead to a disturbance overlaying on the objective image, and the image quality is decreased. The composite photon sieve or special zone plate generates a high-quality point source at the expense of diffraction efficiency [10,11]. Furthermore, deep learning [12,13,14], pixel super-resolution [15,16,17], and differential-interference-contrast [18,19] are used to improve signal noise ratio (SNR), resolution or contrast. However, the sophisticated operation steps far beyond the concise process of single-exposure imaging required.

The point diffraction interferometer is well developed [20,21,22]. A near-ideal spherical wave produced by pinhole diffraction is employed as the reference wave surface [23,24,25,26]. That is a plane wave focused by an objective lens and then across a circular aperture [27,28,29]. When the shape error of the pinhole is controlled within 30 nm with an advanced etching process, the error of the diffraction wave introduced by the pinhole shape is at the magnitude of 10^−4^ λ [30]. The generated source consists of a transmitted wave and a diffracted wave. The effect of the transmitted wave can be neglected when the diameter of the pinhole is not much larger than the pixel size. The point source satisfies the theory of Fresnel diffraction. A well-distributed Fresnel-diffracted spot is filled with the image sensor, and the influence of the machining error could be shifted out of the field of view.

Moreover, an inverse Fresnel algorithm is utilized to reconstruct the objective image. The pinhole-array is arranged in a regular pattern with different sizes so as to match the suitable size easily. Therefore, the structure of DIH is maintained and the sharpness of the objective image is improved.

## 2. Theoretical Analysis of Light Wave Transmission and Reconstruction

### 2.1. Pinhole Diffraction

In original pinhole DIH, an object is illuminated by a spherical wave of wavelength λ, emanating from a pinhole. The object is typically placed a few thousand wavelengths away from the point source. The hologram formed by the un-scattered reference wave and the scattered object wave, is recorded by a large-area image sensor. The light intensity of background-image needs to be restricted at a certain range because both over- and under-exposure lose details, leading to a poor objective image.

To simplify the formula, a point source diffraction is calculated by ignoring the insignificant constant factor. $u_{0}(x_{0},y_{0})$ is the complex amplitude of the object plane, u(x,y) represents the observation plane, and $z_{0}$ is the distance between these two planes. The Huygens–Fresnel diffraction integral formula is given by Equation (1).

(1) $$u_{0}(x,y)=\exp\left\{\frac{jk}{2z_{0}}\left[{\left(x-x_{0}\right)}^{2}+{\left(y-y_{0}\right)}^{2}\right]\right\}$$

Likewise, let us take $\left(x_{r},y_{r},z_{r}\right)$ and $\left(x_{R},y_{R},z_{R}\right)$ and replace the subscripts of $\left(x_{0},y_{0},z_{0}\right)$. The real spherical reference wave diffraction coming from a pinhole is $u_{r}(x,y)$ and the theoretical spherical wave diffraction used in the numerical computation is $u_{R}(x,y)$. The intensity of the hologram is expressed as Equation (2).

(2) $$I_{h}={\left|\beta u_{0}+u_{r}\right|}^{2}={\left(\beta u_{0}\right)}^{2}+u_{r}{}^{2}+\beta u_{0}u_{r}{}^{*}+\beta u_{0}{}^{*}u_{r}$$

where *β* is an amplification factor, *β* = 1, while the reference light is a plane wave. The fourth term $\beta u_{0}{}^{*}u_{r}$ is the conjugate twin image of the third term and will be proportionately reduced in the reverse numerical calculation. Herein, we are only interested in the third term $\beta u_{0}u_{r}{}^{*}$, which encodes the objective image. Taking a spherical reconstruction into account, the wave $u_{3}=u_{R}\beta u_{0}u_{r}{}^{*}$ can be developed as Equation (3).

(3) $$\begin{array}{l} u_{3}=A_{3}\exp\left[\frac{jk}{2}\left(\frac{1}{z_{R}}+\frac{1}{z_{0}}-\frac{1}{z_{r}}\right)\left(x^{2}+y^{2}\right)\right]\\ \;\;\;\;\;\;\;\;\;\;\;\times \exp\left\{-jk\left[\left(\frac{x_{R}}{z_{R}}+\frac{x_{0}}{z_{0}}-\frac{x_{r}}{z_{r}}\right)x+\left(\frac{y_{R}}{z_{R}}+\frac{y_{0}}{z_{0}}-\frac{y_{r}}{z_{r}}\right)y\right]\right\} \end{array}$$

At $A_{3}=\beta A_{R}A_{0}A_{r}{}^{*}$, $A_{0}=\exp\left[jk\left(x_{0}^{2}+y_{0}^{2}\right)/2z_{0}\right]$. $A_{R}$ and $A_{r}$ are similar to $A_{0}$. We often assume that the real value of the spherical wave is consistent with the theoretical value, $u_{R}(x,y)=u_{r}(x,y)$. $u_{3}$ can be rewritten as Equation (4):

(4) $$u_{3}=A_{3}\exp\left[\frac{jk}{2z_{0}}\left(x^{2}+y^{2}\right)\right]\;\times \exp\left[-jk\left(\frac{x_{0}}{z_{0}}x+\frac{y_{0}}{z_{0}}y\right)\right]$$

In this case, the double integral of $u_{3}$ reconstructs the objective wave $u_{0}$. Conversely, if the real reference wave does not conform to diffraction theory $u_{R}(x,y)\neq u_{r}(x,y)$, errors will cause a blurred image of objective reconstruction.

The Fresnel zone construction is used to analyze the diffraction pattern of the circular aperture in the observation plane, which contains alternant and concentric rings of light and darkness. Dark spots cannot be regarded as a reference light. This means that only the central bright spot, namely Fresnel-diffracted spot, is an ideal spherical source. Referring to the formula sinθ≈1.22λ/D, the diffraction angle is inversely proportional to the diameter of aperture at constant wavelength.

### 2.2. Point Diffraction

Introducing a point diffraction into DIH instead of the traditional generation method of a spherical wave, the pinhole diameter a, the converging beam number *F*, and the effect on light intensity *I*, and error *PV* are analyzed. We consider the incident plane wave $U_{0}=1$, then the optical field after the converging lens is ${U^{\prime }}_{0}(x_{0},y_{0})$.

(5) $${U^{\prime }}_{0}(x_{0},y_{0},z_{0})=\mathrm{circ}\left[\frac{\sqrt{\left(x_{0}{}^{2}+y_{0}{}^{2}\right)}}{D/2}\right]\exp\left[-\frac{jk(x_{0}{}^{2}+y_{0}{}^{2})}{2f}\right]$$

where *D* is the diameter of the converging lens, and *f* is the focal length, *F* = *f*/*D*. According to the wavelet superposition principle of Fresnel, the light field within the lens is divided into several secondary wavelet sources, the plane of the pinhole can be expressed as the following Equation (6).

(6) $$U_{1}(x_{1},y_{1},z_{1})=\frac{1}{j\lambda }\iint_{\Sigma }{U^{\prime }}_{0}(x_{0},y_{0})\frac{\exp(jkl_{1})}{l_{1}}d\sigma_{1}$$

With $l_{1}=\sqrt{{\left(x_{1}-x_{0}\right)}^{2}+{\left(y_{1}-y_{0}\right)}^{2}+z_{1}^{2}}$ and the integration proceeds within the pupil radius, the distribution of the wave front on the rear surface of the pinhole is expressed in Equation (7).

(7) $${U^{\prime }}_{1}(x_{1},y_{1},z_{1})=\mathrm{circ}\left[\frac{\sqrt{\left(x_{1}^{2}+y_{1}^{2}\right)}}{a/2}\right]U_{1}(x_{1},y_{1},z_{1})$$

The wavelet superposition principle of Fresnel is applied again to calculate the diffraction from the circular pinhole. The plane of observation can be written as Equation (8).

(8) $$U_{2}(x_{2},y_{2},z_{2})=\frac{1}{j\lambda }\iint_{\Sigma }{U^{\prime }}_{1}(x_{1},y_{1},z_{1})\frac{\exp(jkl_{2})}{l_{2}}d\sigma_{2}$$

With $l_{2}=\sqrt{{\left(x_{2}-x_{1}\right)}^{2}+{\left(y_{2}-y_{1}\right)}^{2}+z_{2}^{2}}$, the light intensity ratio of the pinhole to the total of the focal surface is expressed in Equation (9).

(9) $$t_{I}=\frac{\sum_{\sqrt{x_{2}^{2}+y_{2}^{2}}\leq \tan\theta z_{2}}{\left|U_{2}(x_{2},y_{2},z_{2})\right|}^{2}}{\sum_{\infty }{\left|{U^{\prime }}_{0}(x_{0},y_{0},z_{0})\right|}^{2}}$$

Herein, we only consider the central bright spot, and the effective range of light intensity is $\sqrt{x_{2}^{2}+y_{2}^{2}}\leq \tan\theta z_{2}$. The phase value of a point within the plane of observation can be obtained using the inverse tangent function of Equation (10).

(10) φ=tan−1[Im(U2)/Re(U2)]

where Im (*U_2_*), Re (*U*_2_) represent the imaginary part and the real part, respectively. The wave-front error can be equivalent to the distance between the edge of a diffractive wave and a standard spherical wave. We consider that the optical path difference is no more than a wavelength, and the wave-front error can be expressed as Equation (11).

(11) $$\Delta Er=\lambda (\varphi_{Q}-\varphi_{P})/2\pi$$

With $Q=U_{2}(0,0,z_{2})$ at the vertex of the standard spherical wave and $P=U_{2}(x_{p},y_{p},z_{p})$ at the edge of the diffractive wave. $\varphi_{Q}$ and $\varphi_{P}$ represent the phases of two points Q and P, respectively.

According to Equations (10) and (11), a digital simulation is taken to analyze the pinhole diffractive intensity and wave-front error in *a* = 1~10 μm, λ = 532 nm and *F* = 4, 2, 1, 0.5.

**Figure 1:**
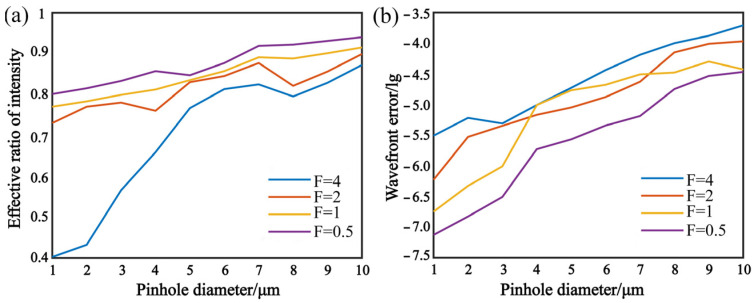
Analysis of pinhole diffractive intensity and wave-front error. (**a**) Effective ratio of intensity diffracted by pinhole. (**b**) Wave-front error of diffractive wave.

Figure 1a,b shows that both of effective ratio and wave-front error increase rapidly with the diameter of the pinhole and decrease with *F*. The wave-front error is smaller than 10^−3^ λ as the diameter of the pinhole is decreasing. Thus, a point diffraction interferometer will generate an approximate ideal spherical wave.

### 2.3. Two-Step Converging Spherical Wave Diffraction

**Figure 2:**
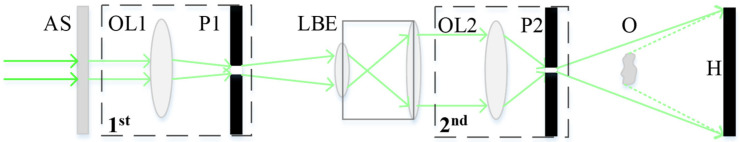
Two-step diffractive path. AS: attenuation slice. OL: objective lens. P: pinhole. LBE: laser beam expander. O: object. H: hologram.

We set $U_{0}=1$ in the previous section, but the quality of a plane wave directly affects the final generated spherical wave. Moreover, the objective lens requires a minimum entrance pupil diameter. A pre-stage point diffraction and laser beam expander are added in our optical path, as shown in Figure 2.

Herein, a point diffraction has two steps. First, OL1 has a low concentration ratio and the diameter of P1 is more than 10 µm. A bright Fresnel-diffracted spot is formed and subsequently amplified and collimated by a laser beam expander. Second, OL2 has a high concentration ratio and the diameter of P2 is less than 10 µm. The hologram is formed by the transmitted spherical wave and the scattered objective wave. Otherwise, an attenuation slice is used to control the laser intensity avoiding over-exposure.

We indicate the recorded hologram by *I*_h_ (r) with the space vector from the point source to the plane of the image sensor r. In order to eliminate the dirty spot over the image sensor and the phase envelope caused by the spherical wave, a contrast hologram is employed to restore the object.

(12) $$I_{c}(r)=I_{h}(r)-I_{o}(r)$$

where *I*_o_ (r) is the background without an object. $r=|r|=\sqrt{x_{i}^{2}+y_{i}^{2}+z_{i}^{2}}$, *x_i_*, *y_i_* are the coordinate positions of the pixel, respectively, *z_i_* is the distance between point source and plane of image sensor. Herein, the coordinates of the pinhole are ignored because the diameter of the pinhole is several microns smaller than the size of the sensor.

We indicate the reconstructive result by K(r) which is a complex amplitude distribution. The recorded hologram is multiplied by the reference light $u_{r}(r)$. K(θ)=(1+cosθ)/2 represents the inclination factor. The integral within the range of the pinhole can be expressed as Equation (13).

(13) $$K(r)=\frac{1}{j\lambda }\iint_{\Sigma }I_{c}(r)u_{r}(r)\frac{\exp(-jk|r-x|)}{\left|r-x\right|}K(\theta )d\sigma$$

**x** denotes the space vector from the point source to the object. $\cos\theta \approx z_{i}/r$. |r−x| cannot be calculated directly by a computer program. A Taylor series expansion is applied to unfold and take the first two terms as follows.

(14) $$\left|r-x|\approx r(1-r•x/r^{2}\right)$$

where $1-r•x/r^{2}$, we take the first term *r* as the denominator. Substituting these parameters into Equation (13), it can be expressed as Equation (15).

(15) $$K(r)=\frac{1}{2j\lambda }\iint_{\Sigma }\left\{\left[I_{h}(r)-I_{o}(r)\right]\frac{1}{r^{2}}\left(1+\frac{z_{i}}{r}\right)\right\}\exp\left(\frac{-jk}{r}r•x\right)d\sigma$$

Here, the complex amplitude distribution of a plane perpendicular to the optical axis can be reconstructed. The result is sensitive to *r*, namely the relative parameter *z_i_*. By changing the distance, a series of two-dimensional planes are obtained and stacked in a stereogram. Furthermore, the distance can be divided into any small sections by a computer program, but the lateral resolution is limited by the pixel number *N* and pixel pitch Δ*x* of the image sensor. The maximum diffractive angle is $\tan\theta =N\Delta x/(2z_{i})$, NA=sinθ.

(16) $$NA=N\Delta x/\sqrt{{\left(2z_{i}\right)}^{2}+{\left(N\Delta x\right)}^{2}}$$

For convolution and angular spectrum reconstruction algorithms of a plane wave, the pixel resolution of the reconstructed image is $\Delta x_{i}=\Delta x$. To satisfy the Nyquist sampling criteria, the object must be far away from the image sensor to ensure that the finest interference fringes can be digitally recorded by an image sensor $\Delta x_{i}=2\Delta x$. Taking the magnification *β* into account, the pixel resolution is $\Delta x_{i}=2\Delta x/\beta$. This makes it flexible in balancing the field of view and resolution with the same magnification as traditional optical microscopy. The lateral resolution is calculated with Equation (17).

(17) $$\Delta \delta =\frac{\lambda }{2NA}\approx \frac{\lambda z_{i}}{N\Delta x}$$

In conclusion, a convergent beam is diffracted at a circular aperture. The ratio of focal length to pupil diameter *F* and the diameter of pinhole α determines the diffractive area. Light before focal point is the converging spherical wave, hence the pinhole must be placed in front of the focal point. A well-distributed spherical wave is generated by adjusting the axis-distance between the pinhole and the objective lens to set a suitable place. The machining error of the pinhole can be shifted to the edge of the image which is negligible for reconstruction.

## 3. Experiments and Results

### 3.1. Experimental Set-Up

**Figure 3:**
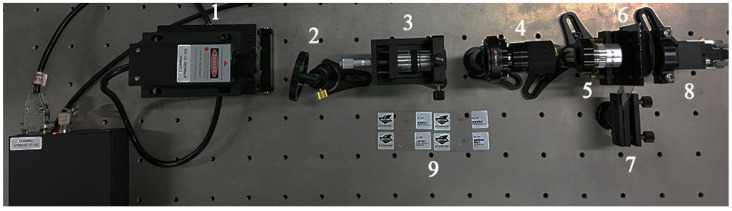
Experimental set-up. (1) Laser-532 nm. (2) Roller attenuator. (3) Spatial filter. (4) Laser beam expander (Sigma LBE-5). (5) Microscope objective (Sigma EPLE-20, *F* = 1.25). (6) Pinhole array. (7) Sample clamping. (8) Camera (Sony IMX183, 5488 × 3672 pixels with 2.4 µm pixel pitch). (9) Biological samples.

Laser coherence is required over the measurement range and a single longitudinal mode solid-state laser is utilized. A linear gradient density filter controls the intensity of light. The spatial filter, as the first step, consists of a microscope objective ×40 and a 304# stainless steel with a pinhole of 40 μm. The long working distance of the microscope objective allows enough space for installing a pinhole. The details are shown in Figure 3.

As shown above, manual focus is difficult at a micron scale without a high-precision physical structure. The precision of pinholes made by machining is limited. We used a 20 × 14 pinhole-array (about 0.5 mm apart) instead of a single circular aperture. It was fabricated by the electron beam writing with a high accuracy work-stage where the substrate is SiO_2_ and chromium is plated on the surface. The vertical direction of the pinhole array is used for coarse-tuning because of a large difference of pinhole diameter, and the horizontal direction is employed for fine-tuning with minor difference.

**Figure 4:**
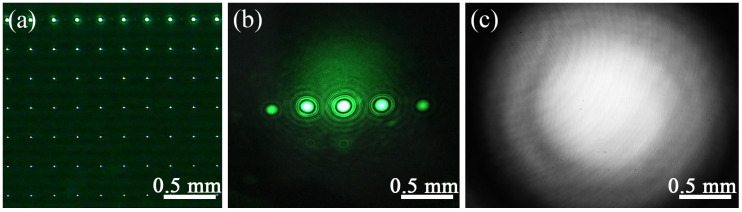
Spherical wave acquisition. (**a**) Pinhole-array. (**b**) Closing the focus. (**c**) A well-distributed diffracted spherical wave.

As shown in Figure 4a, pinholes with the largest diameter are utilized first for a rapid positioning. In this case, the size of a pinhole is beyond the converging spherical wave, a scattered bright circular spot displays on the screen, and the Fresnel diffraction is faint. With coarse-tuning, the intensity of the diffracted spherical wave changes weakly and the area of the center circular spot expands gradually until the center circular spot turns to dark suddenly, as demonstrated in Figure 4b. Fine-tuning shifts the influence of pinhole uncertainty to the edge of the image sensor and then a well-distributed diffracted spherical wave is generated (Figure 4c).

### 3.2. Microscopic Experiments

The measurement process includes four steps: first is the background image (not required for a repeat operation), the second is the interference fringe pattern, the third is a contrast hologram subtracted automatically in a computer program, and the last is the reconstruction procedure.

**Figure 5:**
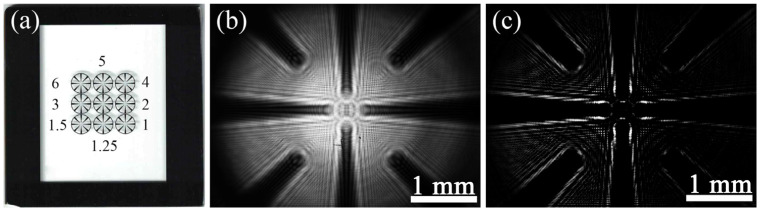
Hologram of resolution board. (**a**) Resolution board (µm). (**b**) Original hologram. (**c**) Contrast hologram.

The resolution board is arrayed in equal proportion as shown in Figure 5a. Three horizontal and vertical lines are in the center, surrounded by the guide lines. After moving the sample to the center of a spherical wave, a hologram (Figure 5b) is collected by the image sensor. Referring to Equation (12), the contrast hologram (Figure 5c) is obtained by subtracting the background image. Moreover, a few fixed dirty spots from the dust are removed from the contrast hologram. The distance between point source and sample is 14 mm, *z_i_* is 44 mm, β≈3.14. 3600 × 3600 pixels are used to restore the object, and the theoretical resolution is 2.71 μm with the field of view 2.75 mm × 2.75 mm.

**Figure 6:**
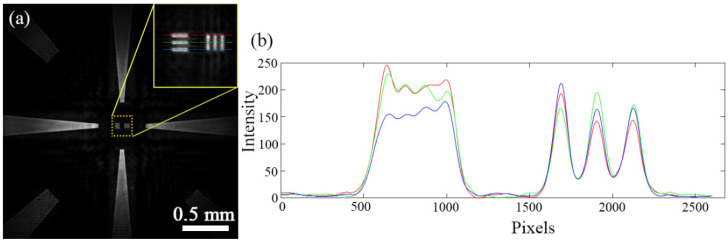
The 10 μm line full-scale microscopy, 3000 × 3000 pixels (2.4 μm). (**a**) Reconstructive image and ×5 magnification of central lines (yellow square). (**b**) Intensity curve (red, green, blue lines).

The 10 μm lines are clearly displayed in Figure 6a. A high-precision spherical wave results in an ideal reconstruction without any image processing. Meanwhile, the twin image is magnified and disappears in background noise. Lines can be clearly identified where the length of lines is five times of the width. Here, we also find a regular distribution of conjugate images near these lines because *β* < 5. Although the intensities of three curves are different, the full width at half maxima are almost the same as in Figure 6b. In conclusion, we believe that it is effective to improve the image quality by improving the spherical wave quality.

**Figure 7:**
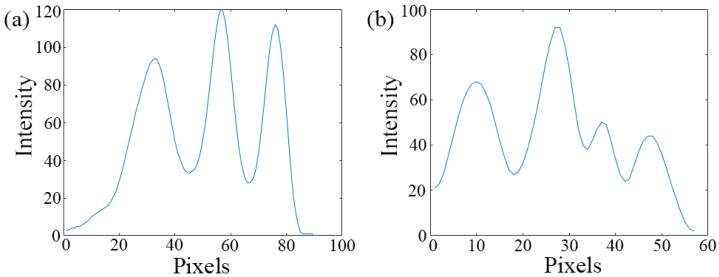
Full-scale microscopy, 3000 × 3000 pixels (2.4 μm), ×10 magnification of central lines and observing the vertical lines with cross-cutting. (**a**) The 5 µm line width. (**b**) The 3 μm line width.

We use the 5 µm and 3 µm line widths to test the resolution of the imaging system. The 5 µm line width is able to be fully identified, but the full width at half maxima is inconsistent (Figure 7a). There are four peaks in Figure 7b, and the 3 µm line width is difficultly be recognized. Due to approaching the limit of resolution and system interference, we consider that the actual resolution of the imaging system is consistent with the theory of resolution.

**Figure 8:**
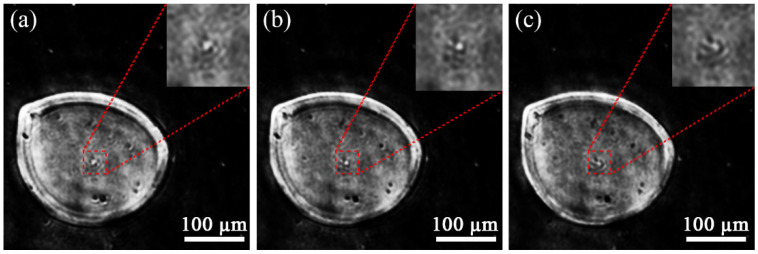
Reconstruction of mosquito eggs at different distances (×5 magnification): (**a**) 13.5 mm, (**b**) 14 mm and (**c**) 14.5 mm.

Biological samples such as egg cells have a certain thickness, and different reconstructive distances will yield different results. In digital reconstruction, while we change the distance from point source to the sample (mosquito eggs), different results are obtained, as shown in Figure 8. The front surface has a wider outline (Figure 8a), the middle layer in Figure 8b is sharper in the center of the egg cell. The aperture is deeper on the rear surface (Figure 8c). Thus, our reconstruction algorithm is sensitive to the distance as analyzed in the previous section.

**Figure 9:**
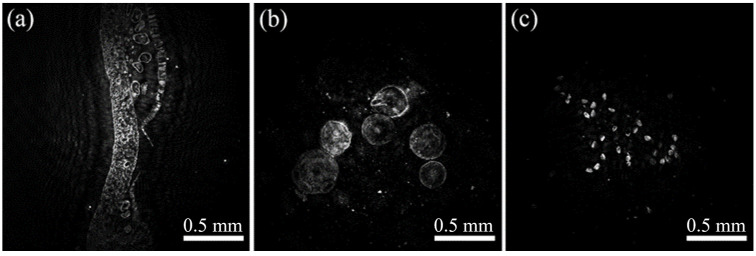
Biological samples. (**a**) Fern spore. (**b**) Volvox. (**c**) Pollen.

Finally, three biological samples with different sizes are utilized to test our DIH. Figure 9a shows a fern spore which displays the imaging of organization slices. The interference fringes are dense, and the result presents the outline directly, but the details are not sufficiently clear to be recognized. Figure 9b shows the volvox with 0.5–1.5 mm diameter, which has a clear plant morphology and characteristics. Figure 9c shows the pollens with a diameter of 4–8 µm. They approach the resolution limit that a few spots mix in the background.

As we discussed, a point diffraction, a converging spherical wave diffracting at a circular aperture, generates a well-distributed diffracted spherical wave. The quality of the reconstructed image can be directly improved, even with a conventional holographic restoration algorithm. A pinhole array with various sizes instead of a specific size design makes it easy to adjust manually. Moreover, single-exposing without focusing and digital reconstructive process can be applied in real-time biological imaging.

## 4. Conclusions

We proposed a method to improve the reconstructive quality of a digital hologram using an approximately ideal spherical wave as a point source. The pinholes of the 1st diffraction are regular metal holes which can be purchased from the market, and the pinhole arrays of the 2nd diffraction are processed by laser direct-writing which is a conventional means of mask processing. Compared with the virtual hole, the diffractive optical element and synthetic aperture methods, a two-step converging spherical wave diffracted at a circular aperture is easy to realize and low cost is achieved.

The image sensor is filled with a well-distributed Fresnel diffracted spot. The tilt factor cannot be neglected with the increase of diffraction angle. The rapid measurement process is obtained due to the single-exposing without focusing. The large field of view and high resolution make it applicable in living cell microscopy. However, the initial studies indicated that the diffracted spherical wave suffers from the effect of a culture medium. Only in pure water, microbes’ activities can be observed effectively.

Furthermore, the numerical aperture of pinhole diffraction is finite. The Fresnel zone plate can provide a larger diffractive angle, but the influence of other diffracted orders needs to be resolved. Image processing can improve the resolution and quality. All our reconstructive results are virgin images. The sub-pixel algorithm, multiple low-resolution pictures composed of a high-resolution image, is a typical method for enhancing resolution that sacrifices the time of measurement. 3D reconstruction is possible because the reconstructive data are sensitive to the distance. Finally, the microscopy system can be used in detection of micro-optical elements, biological recognition, and path tracking of plankton, etc.
